# Effect of the level of manual performance disability caused by exposure to vibration among sailors working on sailing speed vessels

**DOI:** 10.1186/s12891-022-05448-w

**Published:** 2022-05-30

**Authors:** Hamid Saeidnia, Reza Esmaeili, Mohammad Babamiri, Farideh Pourtaghi, Soheil Hassanipour, Gholamhossein Pourtaghi

**Affiliations:** 1 Marine Medicine Research Center, Baqiyatallah University of Medical Sciences, Tehran, Iran; 2 Department of Ergonomics, School of Public Health, Hamadan University of Medical Sciences, Hamadan, Iran; 3 Faculty of Medicine, Shahid Beheshti University of Medical Sciences, Tehran, Iran; 4 Gastrointestinal and Liver Diseases Research Center, Guilan University of Medical Sciences, Rasht, Iran; 5 Health Research Center, Life Style Institute, Baqiyatallah University of Medical Sciences, Tehran, Iran

**Keywords:** Vibration, Grip, Hand disability, Sailing speed vessels

## Abstract

**Background:**

hand-arm vibration is one of the typical annoying physical factors. Hand-arm vibration syndrome (HAVS) is a disorder caused by vibrating working tools which vibrate hands beyond the threshold. Long-term HAVS may result in damage to blood vessels, chronic numbness in the fingers, bone injury, and muscular weakness. People are exposed to high-rate noise vibration in a variety of situations, including vessel employment and operating in tiny boats. Moreover, the extant study was conducted to examine manual function disability levels caused by Sailing Speed Vessels (SSV) vibration.

**Methods:**

The extant study was quasi-experimental research in which, 52 male sailors in SSVs were chosen as the experimental group, and 27 office personnel were selected as the control group. The demographic factors questionnaire, DASH questionnaire, grip and pinch strength tests, the neurosensory exam, and the skill-dexterity test were all employed in this study. SPSS23 software was used to analyze the data.

**Results:**

The findings suggested that the experimental group experienced greater vibration disorder symptoms than the control group. Because the experimental group had a higher score, the individuals experienced poorer circumstances in terms of arm, shoulder, and hand impairment as compared to the control group. The mean grip strength of hands and fingers in two hands of the experimental group was lower than the control group (*P* < 0.05). There was a statistically significant relationship among grip strengths of both experimental and control groups (*P* < 0.05). There was a reduction in skill and dexterity of both dominant and non-dominant hands of members in the experimental group. According to the statistical tests, there was no significant association between dominant (*P =* 0.001) skills and non-dominant (*P =* 0.010) hands in experimental and control groups. There was not also any significant relationship between skill and dexterity of both hands (*P =* 0.001) and the dominant hand tweezer test (*P =* 0.001) in two experimental and control groups. There was a statistically significant association between experimental and control groups in terms of assembly skill and dexterity (*P =* 0.482).

**Conclusion:**

Individuals who are at risk of vibration experience less physical and sensory function. DASH score, grip strength, skill, and dexterity could predict the reduction in physical function disability.

## Background

The vibration occurs when the body is exposed to a vibrating surface [1]. The hand and arm are the most common areas exposed to such vibrating surfaces [2]. Vibration is caused by some tools, such as drills, cutting machines, milling machines, types of internal combustion engines, pneumatic tools, road, and marine vehicles, as well as machines with moving parts [3]. Vibrations generated by road and marine vehicles and machinery are a frequent source of physical danger in the workplace [3]. Hand-arm vibration syndrome (HAVS) is a condition induced by vibrating working instruments that exceed the threshold of vibration in the hands [4]. Around 25 million employees in Europe are exposed to workplace vibration, which may result in serious damage [5]. In the USA, 2 million workers are at risk of hand and arm vibration; of them, 50% experience HAVS [5]. Moreover, 72,000-144,000 HAVS cases were reported in Canada, 2017 [6].

The main vibration sources include variable and oscillating forces on the impeller, transfer forces from the axes induced by irregular eddy currents, change of dynamic pressure field around the body, the existence of rotating unbalanced parts, non-coaxial, erosion, and similar cases in machines (e.g., in sea vessels) [7, 8]. The mentioned vibrations may occur in the whole body of the vessel or some parts locally [8, 9].

These factors will have destructive effects on offshore users. Most of these effects in the long run will cause serious defects for floating users [10]. These include damage to the nervous and vascular systems and musculoskeletal disorders [11]. The complications caused by the vibration effect on the psychomotor functions are important in terms of safety and health, so they require further assessment and experiments [4]. Long and repetitive contact with relatively severe vibration on a surface with no rapid destructive effect can harm the health in some professions and jobs [12, 13]. Environmentally, vibration causes dysfunction by affecting the vision accuracy or focus when working with control tools and devices [14]. Such effects depend on the frequency and acceleration rate in the vibrating body [15]. In some jobs, especially those requiring accurate collaboration and balance between hands and eyes, skill and accuracy while exposure to average vibration can reduce speed [16]. Through central or environmental processes, severe vibration and oscillating motion of persons may reduce working efficiency or rate of fulfilling job [17]. Stressful, dull, and potentially hazardous oscillation and vibration may impair work performance [13, 18]. According to studies conducted on psychological tests, exposure to vibration may cause some neurosensory disorders, such as numbness, tingling, and fatigue [19, 20]. Moreover, other studies indicated that long exposure to vibration reduces hands strength, skill, and manual dexterity [20, 21].

Hands are one of the most vital extremities of humans help for physical change in the surrounding environment [15]. Regarding their complex and specific musculoskeletal systems, hands allow the person to do various activities and works [2]. Hand dexterity and hand strength (gripping items with palms and fingers) are two important hand actions [22]. The most essential parameters impacting hand function are grip strength and manual dexterity [23]. Force exertion is a physical activity, so excessive force is a physical activity that exceeds the tolerable physiological exertion [24–26]. Medically, the upper extremities’ experiment is done based on observation and subjective aspects although measurement of hands strength and skill provides the physician with quantitative and objective data of hand function [27]. Hands strength and skill are evaluated as a health index in many medical clinics [27, 28]; it is also one of the substantial parameters considered when recruiting applicants in some countries [29].

Vibration is regarded a profession that results in the assessment of the degree of job-related exposure to work, as one of the most significant physical damaging components of the work environment from a health viewpoint [2]. Exposure to the vibration of hands and arms is common among labor workers in the country’s industrial environments and is 10% exposed to vibration [30]. Vibration of the hands and arms over time can lead to neurological, vascular, and musculoskeletal disorders as symptoms of hand tremors [31]. Vibration measurement of the hand and arm is required to assess the risk of exposure to vibration and to determine the amount of radiation emitted in different machines [14].

Despite many studies conducted on the effects caused by vibration in vessels on the different aspects of human functioning, there is no considerable research on the effects of this factor on psychosomatic aspects of humans, such as skill, dexterity, and hands strength of boatmen. On the other hand, the results of this study can be used to expand psychosomatic factors of boatmen to improve their efficiency and productivity. Therefore, the objectives of this study:

1. Measure the daily equivalent acceleration of vibration in SSVs.
2. Determine skill and manual dexterity of Sailors.
3. Determine grip and pinch strength of Sailors hands.
4. Find the relationship between skill and dexterity of experimental and control groups.
5. Find the relationship between grip and pinch strength of experimental and control groups.

## Materials and methods

### Sampling process

The extant study was quasi-experimental research conducted to examine manual disability among boatmen caused by exposure to vibration in 2020. The target population comprised sailors in SSVs who work in southern areas of Iran. 85 male sailors participated, but 79 participants presented their full information to the researchers. In this case, 52 sailor in SSVs were assigned to the experimental group, and 27 office personnel were selected as the control group. In terms of demographic variables, such as age, sex, and job experience, the control and experimental groups were matched. The control group, on the other hand, was not subjected to vessel vibration.

### Implementation process

To reduce confounding circumstances, the data were gathered during the first working shift (morning) and participants were guaranteed that they had no mission before filling out the questionnaire. The effect of vibration on the participants was examined based on the following inclusion criteria: having at least 1 year of employment history, no injury in upper extremities, especially in hands and shoulders in recent months, physical and psychological health. The inclusion criteria were recorded as questions in the demographic questionnaire. Following ethical principles in human research, all participants signed the consent letter after receiving information about the research objectives. Moreover, the following steps were taken:

### Step 1: measuring hand and arm vibration

Hand and arm vibration was measured using the 106SA Svantek vibration meter in three x, y, and z axes according to ISO5349 standard [32]. Vibration assessment was done by summing weighted acceleration obtained from combining three axes and following eq. [33]:

1 $${a}_{hw=\sqrt{a_{hw y}^2+{a}_{hw x}^2+{a}_{hw z}^2}}$$

Equation 1 indicates the vibration acceleration result in which, a_hwy_, a_hwx_, and a_hwz_ show effective acceleration in each axis. The following equation indicates 8-hour vibration acceleration in which T represents the total time of exposure (hr) and T_0_ shows the considered limited time (8 hr) [1].

2 $$A(8)={a}_{hw}\sqrt{\frac{T}{T_0}}$$

Where *a*_*hw*_ is Effective acceleration, *T* is Total exposure time and *T*_0_ is Standard exposure time (8 h).

The vibrations of the hand and arm were measured using finger sensors. The ship’s crew was permitted to grasp the wrist without any change in wrist position or grip force after configuring and managing the device’s connections and choosing the axle. The vibration was measured in three directions consecutively and in such a way that the working conditions in each of three measurements were uniform. The weather was sunny, and the sea was calm during the measurement. Hand and arm vibrations were measured for all participants, and finally the mean number was reported. The vessels were small and medium-speed vessels with a capacity of five to twenty people.

### Step 2: examining demographic data, job and medical records

The questionnaire was adopted from the Institute of Sound and Vibration Research (ISVR) at the University of Southampton, UK, and the Occupational Medicine and Rehabilitation Institute at the University of Trieste, Italy to examine complications of exposure to hand and arm vibration [1]. This questionnaire included personal information, job records, exposure to vibration, vibration complications (arterial, neurosensory, and musculoskeletal), and medical records of the worker.

### Step 3: assessment of hand and arm disabilities

Disabilities of the Arm, Shoulder, and Hand (DASH) questionnaire was used to measure the disability level of individuals when exposed to hand and arm vibration [34]. This questionnaire inquires about the capacity to do particular tasks with the hands. Scores are assigned to respondents depending on their replies [35, 36]. According to the validity and reliability of this questionnaire in Iran, the Content Validity Ratio (CVR) and Content Validity Index (CVI) of the DASH questionnaire were 0.74 and 0.90, respectively. Cronbach’s alpha coefficient of this questionnaire was estimated to be 0.75 [1].

### Step 4: measuring skill, dexterity, and tactile sensitivity

Purdue Pegboard A32020 Model with a reliability interval of 0.76-0.89 was used to measure skill and dexterity [1]. Purdue Pegboard has some holes used by the person to place the metal pieces and washers in respective holes. In this case, the skill and manual dexterity of a person is measured based on the spent time.

Purdue Pegboard includes three tests of the right hand (RH) (dominant hand), left hand (LH) (non-dominant hand), and both hands (BH). Purdue Pegboard consists of a board with 4 cups across the top and two vertical rows of 25 small holes down the center. There are 25 pins in each of the two cups, 40 washers in one cup, and 20 collars in the other. Participants in RH and LH tests must utilize their dominant (right) and non-dominant (left) hands to put more pins in the relevant row in fewer than 30 seconds. These subtests are graded on the total number of pins set by each hand in a certain amount of time.

In multiple BH test, participants use both hands simultaneously to place pins down respective rows within 30 seconds. The score of this subtest is measured according to a total number of pairs of pins within 30 seconds [37].

Eye-hand coordination was used to evaluate the standard, and the O’Connor Tweezer Dexterity test was used to control subtle motion. The latter test is used to measure skilled motions of arm and hand because it is a valid and reliable test of skill. This test comprises a board that contains 100 holes (10 rows with 10 holes each) and one cup of 100 pins. The participant’s dominant hand was requested to use a Tweezer to insert all 100 pins into holes in the quickest time possible. Only one route is scored for the amount of time it takes to place all pins in holes. To reduce the learning effect, each participant was permitted to finish 10 holes across the top of the board. The time (second) required for test completion converts the raw score to a scaled value (standard score in this study) [38].

A neurosensory test and Monofilament kit with a reliability interval of 0.60-0.99 was used to assess tactile sensitivity [1]. Monofilament kits with different numbers were placed on various parts of the hands to perform this test [39].

### Step 5: measuring grip strength of fingers and hands

The grip strength of fingers was evaluated using a Pinch Gauge based on the force in kilograms imposed by the person that pressed the device key. The Jamar hydraulic hand dynamometer was used to measure the maximum grip strength of the hands. In this test, the person puts his/her elbow with 90° degree on the flat surface and imposes the maximum force (in kilograms) by gripping the handle within 10-seconds intervals. Relevant studies have introduced Jamar hydraulic hand dynamometer as a golden standard with relatively good and excellent reliability (ICC -0.92-0.90) that measures the grip strength of hands [1, 40, 41].

### Step 6: data analysis

Data analysis was done through SPSS23 software. The independent sample t-test and chi-square tests were used for data analysis. Odds ratio (OR) and Standardized mean difference (SMD) were calculated from CMA software. A *P-*value of less than 0.05 was considered as significant.

## Results

### Demographic characteristics

This study comprised 85 male sailors participants of which, 79 members were eligible to participate. The mean value (standard deviation) of participants’ age was 32.96 (3.81). Moreover, the mean Work experience of studied participants was more than 5 years. In terms of job, the majority of participants were sailor in SSVs (*n* = 52) in the experimental group, while 27 members were office personnel in the control group. The mean body mass index (BMI) was 25.18 with a standard deviation of 2.12 kg/m^2^. Table 1 reports the results of demographic characteristics of studied sailor in SSVs and control group.

**Table 1:** Mean and SD of age, BMI and work experience of participants

Variables	Group	M	SD	Maximal	Minimal
Age (years)	experimental	34.77	2.65	41	30
	control	32.01	4.00	42	24
Work experience (years)	experimental	10.37	1.94	16	8
	control	8.46	2.76	15	2
BMI (kg/m^2^)	experimental	26.08	1.93	30.49	23.15
	control	24.72	2.08	30.49	20.98

*BMI* Body Mass Index, *M* Mean, *SD* Standard Deviation.

### Vibration exposure status

The mean value of vibration acceleration in three x, y, and z axes was 6.19, 2.68, and 7.54 m/s^2^, respectively. The mean vibration along axis y was less than that along axes x and z. Statistical analyses revealed that the comparable 8-hour acceleration of hand and arm vibration exposure in boatmen exceeded the Iranian standard (2 m/s2) [1]. The measured values of vibration acceleration of SSVs were reported in Table 2.

**Table 2:** Values of vibration acceleration of SSVs

Measurement mode	Measurement of time (S)	Mean Daily Exposure Time (S)	Frequency weight acceleration (m/s^2^)			Resultant XYZ	Daily Vibration Exposure A(8)
			X	Y	Z		
Vessels in stillness	445	5400	3.03	2.6	5.44	6.75	3.37
Vessels moving at low speed (20 Km/h)	362	7200	7.36	1.95	7.92	11	5.49
Vessels moving at high speed (100 Km/h)	312	7200	8.19	3.5	9.26	12.84	5.56
Mean	373	6600	6.19	2.68	7.54	10.19	4.81
SD	67.2	1039	2.77	0.78	1.93	3.12	1.24

*M* Mean, *SD* Standard Deviation.

### Hand and arm disabilities

**Fig. 1:**
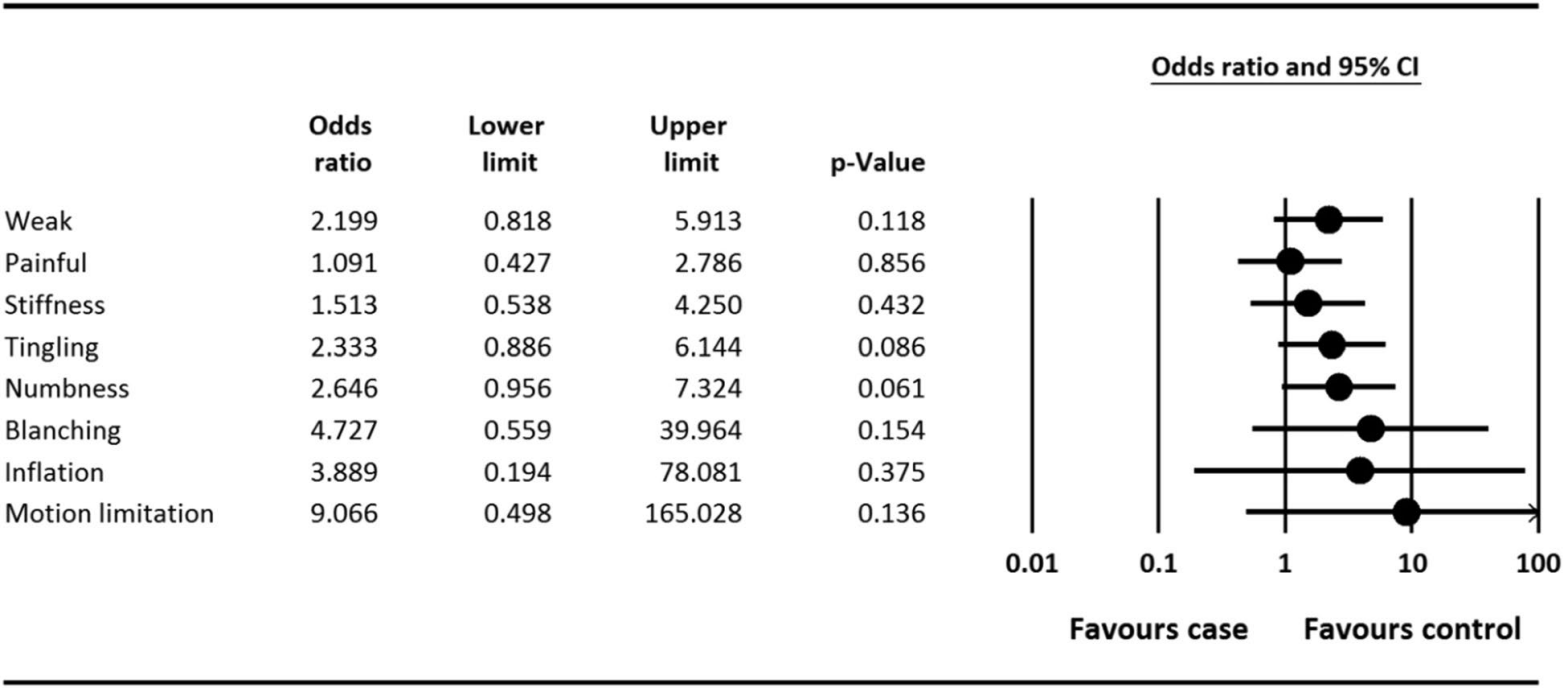
Symptoms of hand and arm vibration syndrome. Odds ratio (OR): OR > 1 indicates increased occurrence of event, OR < 1 indicates decreased occurrence of event

According to results shown in Fig. 1, all of the disorder symptoms caused by vibration, such as the white finger, tingling, numbness, trigger finger, swollen fingers, pain, finger weakness in gripping objects, and hand movement limits were observed in the experimental group compared to control group.

According to the results of statistical tests obtained from the DASH questionnaire, the experimental group (10.57 ± 13.07) obtained a higher score, so they had the worse status of disabilities of the arm, shoulder, and hand compared to the control group (3.42 ± 3.28).

### Grip strength of fingers and hands

**Fig. 2:**
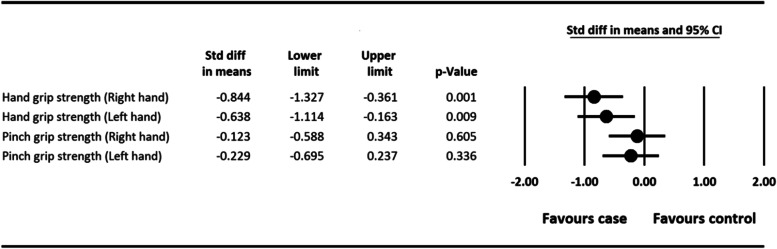
Average strength of hands and fingers of case and control group. Std diff: standardized difference

As seen in Fig. 2, the mean grip strength of hands and fingers of both hands in the experimental group was lower than the control group. According to statistical analyses, there was no link between dominant and non-dominant hand grip strength and DASH questionnaire score in the experimental group (*P* > 0.05), indicating that there was an indirect relationship between hand grip strength and arm, shoulder, and hand disabilities. On the other hand, there was a significant difference between pinch strength of the dominant hand (SMD = -0.848, 95% CI: − 1.327 to − 0.361, *P =* 0.001) and non-dominant hand (SMD = -0.638, 95% CI: − 1.114 to − 0.163, *P =* 0.001) in two experimental and control groups (Fig. 2). However, there was not any significant difference between the strength of the dominant hand’s fingers (SMD = -0.123, 95% CI: − 0.588 to 0.343, *P =* 0.605) and non-dominant hand’s fingers (SMD = -0.229, 95% CI: − 0.695 to 0.237, *P =* 0.336) in two experimental and control groups (Fig. 2).

there was no direct association between grip strength of hand and fingers and personal (demographic) information, so any increase (decrease) in demographic variables did not lead to higher (lower) grip strength of hand and fingers (Table 3).

**Table 3:** The strength of hands and fingers and characteristics of participants

variables		Grip strength		Pinch strength	
		Dominant hand	No Dominant hand	Dominant hand	No Dominant hand
Age	Coefficient	−0.08	−0.09	0.06	0.49
	‌*P-*value*	0.94	0.39	0.60	0.66
Work experience	Coefficient	0.01	−0.05	0.64	−0.07
	‌*P-*value*	0.90	0.65	0.57	0.49
BMI	Coefficient	0.06	0.07	−0.06	0.02
	‌*P-*value*	0.58	0.53	0.57	0.83

*: *P-*value of Pearson correlation coefficient

### Skill, dexterity, and tactile sensitivity

**Fig. 3:**
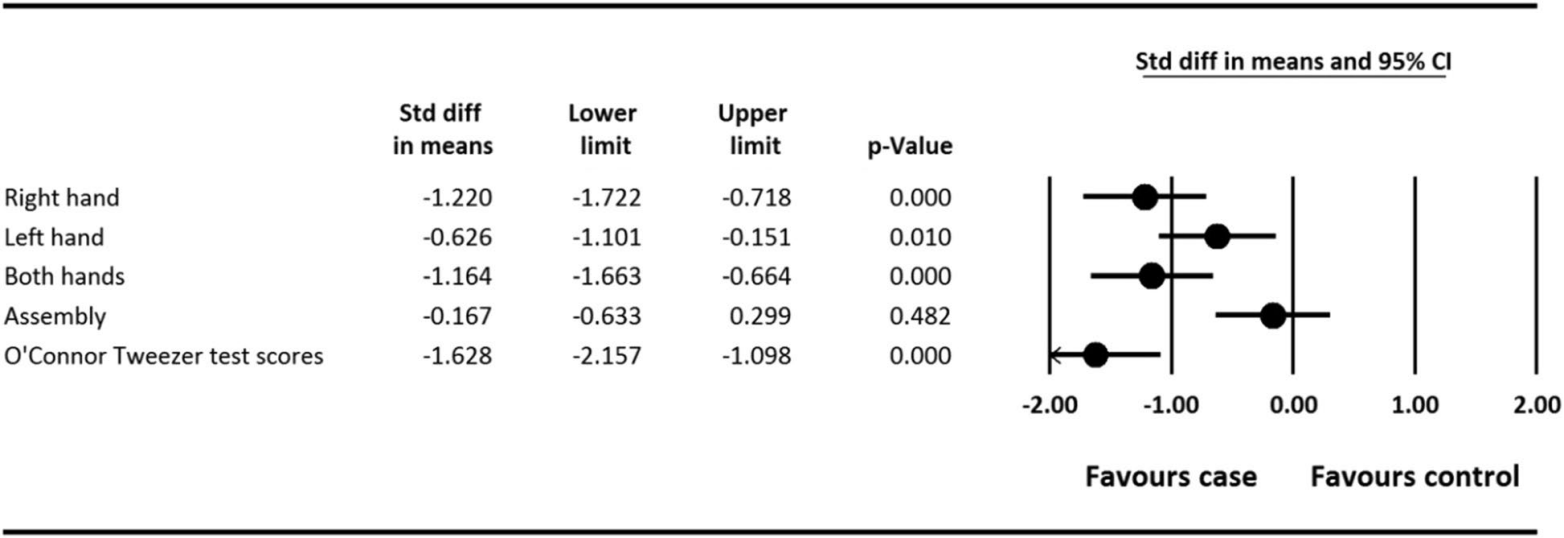
The relationship between skill and dexterity in case and control group. Std diff: standardized difference

As seen in Fig. 3, the skill and dexterity of dominant and non-dominant hands were reduced in the experimental group. There was a significant difference between skill and dexterity of dominant (SMD = -1.220, 95% CI: − 1.722 to − 0.718, *P =* 0.001) and non-dominant hands (SMD = -0.626, 95% CI: − 1.101 to − 0.15, *P =* 0.010) in two experimental and control groups (Fig. 3). There was also a significant difference between skill and dexterity of both hands (SMD = -1.164, 95% CI: − 1.633 to − 0.664, *P =* 0.001) and Tweezer test of the dominant hand (SMD = -1.628, 95% CI: − 2.157 to − 1.098, *P =* 0.001) in two experimental and control groups (Fig. 3). There was not any significant difference between the two experimental and control groups in terms of skill, dexterity, and assembly (SMD = -.167, 95% CI: −.633 to 0.299, *P =* 0.482) (Fig. 3).

Other results of study indicated that tactile sensitivity of the dominant hand (SMD = 0.585, 95% CI: 0.111 to 1.059, *P =* 0.015) in the experimental group was higher than in the control group, and there was a significant relationship between the two groups.

## Discussion

The existing research examined the manual function deficits suffered by boatmen as a result of vibration exposure. According to the findings of an instrumental examination of musculoskeletal issues in both dominant and non-dominant hands, the experimental group had greater complications than the control group. The disorder symptoms caused by vibration, including white finger, tingling, numbness, trigger finger, swollen fingers, pain, finger weakness in gripping objects, and hand movement limits in experimental groups were more than in the control group. Because the experimental group obtained a higher DASH score, this group had worse conditions in terms of disabilities of arm, shoulder, and hand compared to the control group. There is no similar study on S; however, DASH score of the studied population in the extant study was in line with findings of previous studies on individuals who were exposed to vibration [42, 43]. Consistent with the present paper, Buhaug et al. found a high level of upper limb disability in studied patients, and the experimental group obtained a higher DASH score rather than the control group, i.e., disability in the upper limb in the group affected by vibration was higher than the control group [44]. House et al. concluded that individuals affected by vibration experienced disability in their upper extremities [43]. This study indicated a correlation between DASH score and several variables of which, upper extremity pain had the highest effect. Because upper extremity pain is the most prevalent musculoskeletal symptom caused by arm vibration [45], the authors found the highest effect of musculoskeletal factors on disability.

Other findings of the extant study indicated that participants in the experimental group faced more neurosensory complications caused by vibration compared to the control group. This result was matched with findings obtained by Alabadi et al. [1] and Bovenzi et al. [46]. The data collected through questionnaires showed that neurosensory disorders were more prevalent than musculoskeletal and arterial disorders; this finding was consistent with results obtained by Aliabadi et al. [47] and Poorabdian et al. [48]. Unlike our study, Pollard et al. investigated the effect of vibration on grip strength and touch sensation of haul trucks’ drivers and found no significant correlation between touch sensation, grip strength, and-arm vibration, and whole-body vibration [49]. The vibration exposure level of the operators in this investigation was insufficient to have an acute impact, but may have been adequate to cause long-term alterations. This might account for the discrepancy in our findings. Experiment results indicated lower hand skill and strength in the vibration exposure group compared to the experimental group. Aliabadi et al. carried out a study on the level of manual performance disability caused by exposure to hand-arm vibration and found lower grip strength and dexterity among vibration exposure group members [1]. Toibana et al. measured manipulative dexterity in patients with hand-arm vibration syndrome and found less grip strength and dexterity in these patients [50]. Unlike the previous research, Vallejo et al. (2007) examined sailors’ grip strength and determined that sailors have greater grip strength than the control group. The dominant hand was shown to be stronger than the non-dominant hand in this research [51]. The reason for such difference may stem from technological progress in the marine industry in developed countries.

The grip strength of the fingers of both hands was higher in the experimental group. Moreover, there was not any direct correlation between hand/fingers grip strength and demographic characteristics, so an increase (decrease) in demographic variables did not lead to higher (lower) grip strength of hand and fingers. Soori et al. [28] and Liao et al. [52] measured grip strength and BMI of the individuals and found a significant association between these two variables. On the other hand, people with average BMI had higher grip strength. In addition, Mohammadian et al. found a positive and significant relationship between fingers’ strength and BMI [53]. Barbara et al. studied the effect of contextual variables on the grip strength and found no significant correlation between age and grip strength of participants [54]. Further studies indicated that aging led to lower grip strength [55–58]. Mathiowetz et al. concluded that young people aged 25-39 had higher grip strength [56]. Schmidt et al. found that people aged 27-32 had higher grip strength compared to other age groups [59]. Schlüssel et al. examined grip strength in adults and concluded that handgrip strength significantly decreased after 40 [60]. Taekema et al. found that weak grip strength could predict reducing in physical and cognitive disability [61]. Farkkila (1980) carried out a study on vibration-induced neuropathy and found that paraesthesia symptoms, numbness, finger touch sensation, skill, and dexterity of individuals with vibration exposure had worse situations compared to the control group. The results of the respective questionnaire and tests of tactile sensory (Monofilament) and dexterity (pegboard) confirmed the findings [62]. In a study aimed at describing the upper limb disability of Norwegian workers with hand-arm vibration syndrome by Buhaug et al., the workers medical records were reviewed and the DASH questionnaire was completed by them. The results showed that the disability of the upper limbs in these workers was much higher compared to the control group. Also, the mean of DASH score in the case group was 41.2, while this score was 10 in the control group, which means that the inability of the upper limb in the group exposed to vibration is more than the control group. They also found a significant relationship between DASH score and hand grip strength [44]. In the present study, the DASH score in the case group (10.57) was higher than the control group (3.42). However, there was no significant relationship between DASH score and grip strength. It seems that the reason for the difference in the results of the two studies was the difference in the years of exposure to vibration and the difference in the type of jobs between the two studies. The extant study found that the vibration exposure group experienced more musculoskeletal, neurosensory, and arterial complications compared to the control group. In general, vibration exposure decreased the manual function and dexterity of vessel drivers. The current research encountered certain limitations; for example, it was not able to conduct testing before to working a shift and being exposed to workplace vibration in order to compare the findings to those obtained after the shift. Another constraint was large sample size. Therefore, it is recommended to consider these points in further studies.

## Conclusion

The extant study aimed to examine the vibration-induced manual disability of vessel drivers. The results found more musculoskeletal, neurosensory, and arterial complications in the vibration exposure group compared to the control group. The experimental group had lower grip strength and finger skills in both hands rather than the control group. In general, vibration exposure decreased the function level of sailor in SSVs.

Following control measures are recommended to improve staff’s situation and alleviate the effect of vibration:

- Using vibration dampers and absorbers in the body of SSVs.
- Minimizing the time of vibration exposure.
- Using Personal protective equipment against vibration, such as anti-vibration gloves and shoes.

## Data Availability

Due to the request of the participants in the study and the protection of their privacy, we are exempt from disclosing their personal information publically. The datasets used and analysed during the current study are available from the corresponding author on reasonable request.
